# Interactive peer-guided examination preparation course for second-year international full-time medical students: quantitative and qualitative evaluation

**DOI:** 10.3205/zma001203

**Published:** 2018-11-30

**Authors:** Daniel Huhn, Karam Al Halabi, Obada Alhalabi, Christina Armstrong, Alexandra Castell Morley, Wolfgang Herzog, Christoph Nikendei

**Affiliations:** 1 University of Heidelberg, University Hospital for General Internal and Psychosomatic Medicine, Centre for Psychosocial Medicine, Heidelberg, Germany; 2 Ruprecht-Karls-University Heidelberg, Tutor of Heidelberg's Tutorial for International Medical Students (HeiTiMed), Heidelberg, Germany

**Keywords:** international medical students, evaluation, examination preparation course

## Abstract

**Background: **It has been documented that international students face diverse challenges due to language and cultural barriers. International medical students suffer from personal distress, a lack of support and perform poorer than local fellow-students in clinical examinations. It has been documented that international medical students benefit from peer-led tutorials in their first year. We investigated the effectiveness of a tutorial offered for international medical students in their second year.

**Methods: **A peer-guided examination preparation course with interactive elements for second year international medical students was designed, learning objectives were defined. Two evaluations were undertaken: In a quantitative assessment, students were asked to fill out five multiple-choice-questions at the beginning of every session of the tutorial (pre-test) as well as to participate in a post-test at the end of the semester in which all former multiple-choice-questions were re-used. Using a qualitative approach, participants were asked for their thoughts and comments in a semi-structured interview at the end of the semester.

**Results:** International students (N=12) showed significantly better results in the post- than in the pre-test (t(11)=–8.48, p<.001, d=1.95). Within the interviews, international students (N=10) reported to have benefited from technical and didactic, as well as social learning experiences. The individual lectures students were asked to contribute were discussed controversially.

**Conclusion: **Our peer-guided tutorial for second year international medical students is an effective and well accepted possibility to prepare these students for examinations.

## Zusammenfassung

**Hintergrund: **Es ist bekannt, dass internationale Studierende aufgrund bestehender sprachlicher und kultureller Hürden vor verschiedenartige Herausforderungen gestellt werden. Internationale Medizinstudierende leiden unter persönlichem Stress sowie unzureichender Unterstützung und zeigen in klinischen Prüfungen schlechtere Ergebnisse als einheimische Kommilitonen. Es konnte gezeigt werden, dass internationale Medizinstudierende im ersten Studienjahr von studentengeleiteten Tutorien profitieren können. In der vorliegenden Studie wurde die Wirksamkeit eines Tutoriums für internationale Medizinstudierende im zweiten Studienjahr untersucht.

**Methodik:** Ein studentengeleitetes Tutorium mit interaktiven Elementen für internationale Medizinstudierende im zweiten Studienjahr wurde konzipiert und Lernziele wurden definiert. Zwei Evaluationen wurden dabei vorgenommen: In einer quantitativen Erhebung waren Studierende aufgefordert, zu Beginn jeder Tutoriumssitzung fünf Multiple-Choice-Fragen zu beantworten (Prä-Test) und außerdem, am Ende des Semesters an einem Post-Test teilzunehmen, in welchem alle vorherigen Multiple-Choice-Fragen erneut verwendet wurden. In einem qualitativen Ansatz wurden freiwillige Teilnehmer am Ende des Semesters bzgl. ihrer Gedanken und Anmerkungen in halbstrukturierten Interviews befragt.

**Ergebnisse:** Internationale Studierende (N=12) zeigten signifikant bessere Ergebnisse in den Post-Tests als in den Prä-Tests (t(11)=–8,48, p<,001, d=1,95). Im Rahmen der Interviews berichteten die internationalen Studierenden (N=10), im Tutorium von technisch-didaktischen sowie sozialen Lernerfahrungen profitiert zu haben. Die individuellen Vorträge, welche die Studierenden gebeten worden waren zu halten, wurden kontrovers diskutiert.

**Schlussfolgerung:** Unser studentengeleitetes Tutorium für internationale Medizinstudierende im zweiten Studienjahr ist eine effektive und gut angenommene Möglichkeit, diese Studierenden auf Prüfungen vorzubereiten.

## Introduction

In 2015, a total of 321,569 international students were enrolled at German higher education institutions. Compared to 2005, this figure has increased by 31% or approximately 75,000 students [1]. The percentage of international students in Germany’s total student population has increased to 11.9%, of which 8.7% are so called “Bildungsausländer” and 3.2% are “Bildungsinländer” [2]. “Bildungsinländer” are students of other nationalities who have obtained their higher education entrance qualification either in Germany or a German school abroad. “Bildungsausländer” are students of other nationalities, who have obtained their higher education entrance qualification outside Germany. China is the most important country of origin with more than 30,000 students studying in Germany, followed by India, Russia, Austria and France as frequent countries of origin [2]. The largest proportion of international students are enrolled in engineering (23%), followed by language and cultural studies (22%) [2]. 

Within the fields of medicine and health sciences, 13,100 international students were enrolled at higher education institutions in the year 2015, which accounts for 10.6% of all medical and health science students. Regarding medical education, it has been documented that international medical students more often suffer from personal distress [3] as well as report of reduced quality of life [4], insufficient support [5] and a loss of social contacts [6] during their studies. Probably due to existing language and cultural barriers [7], [8], international medical students perform worse in written, oral and practical examinations [9], [10], [11], [12], [13], show a prolonged duration of study [14] as well as higher drop-out rates [9], [15]. However, at the very beginning of their studies, international medical students seem to go through their most critical period, especially when they are the most confronted with cultural barriers [16].

Peer-assisted learning (PAL) is a well-established as well as effective teaching method within medical education [17], [18]. PAL can be defined as “the development of knowledge and skill through active help and support among status equals or matched companions” [19]. For international students, PAL represents a well-accepted learning experience [20]. During the PAL processes, tutors’ and participants’ cognitive and social congruence seem to raise learning efficacy [21]. For example, if tutors are of an international background themselves, they can operate as authentic role models. Furthermore, as they are themselves students, they are more likely to use comprehensible language and foster a permissive learning atmosphere [20]. However, to the best of our knowledge, no studies concerning the effectiveness of peer-guided courses for international students have been conducted so far.

The purpose of this study was to thus test the effectiveness of a newly designed peer-guided course for international medical students (Heidelberg Tutorial for international Medical students – HeiTiMed) in their second year. We hypothesised that participation in the course would show an impact

1. on the pre-post-comparison of multiple-choice-questions as well as
2. on students’ satisfaction.

## Methods

### Aim of the study and study design

The study aimed to determine whether a voluntary, peer-guided course for second year (third and fourth semester) international medical students is an effective method to prepare participants for the final examination at the end of the third semester. It is a prospective study with quantitative as well as qualitative measures. Participants were asked to answer five multiple-choice questions, as a pre-test, at the beginning of each session of the tutorial assessing topics related to the learning objectives of the following session. At the end of the semester, they were asked to write a post-test during the last session of the tutorial. The post-test consisted of the same multiple-choice questions that students had already answered in pre-tests during the semester. Following this test, students had to write a mandatory multiple-choice examination covering contents of the whole semester as a part of the curriculum; the goal of the tutorial was to prepare for this examination. At the end of the semester, semi-structured interviews were conducted with a selection of participants to consider their thoughts and comments.

#### Definition of learning objectives

As previously reported [20], we conceived a tutorial for first year (first and second semester) international students at our medical faculty, that was launched in the winter term 2013/2014 [20]. This tutorial aims to equip participants with knowledge and skills for first year examinations and to familiarize participants with living and studying in Germany. As second year students have mostly become more accustomed to a student-life abroad the here presented tutorial newly developed for second year students was designed to emphasize optimal learning techniques. Following Kern`s principles of curriculum development [22], the course was designed to

1. deliver essential knowledge for the examinations of the third semester; and
2. to enable the acquaintance with holding a short presentation on subject related contents.

General learning objectives were defined as follows:

1. Knowledge: by the end of the course, participants will have achieved cognitive proficiency in the relevant topics of the third semester.
2. Attitude: participants will also have gained confidence in their examination-related abilities. Furthermore,
3. skills: participants will have improved their abilities in presenting medical-related contents in German language in front of others at the end of the course.

#### The third semester at the Medical Faculty at the University of Heidelberg

Within the second year of medical education at the Heidelberg University Medical Faculty, the third semester integrates the subjects of physiology, biochemistry and histology. Students gain proficiency in profound disciplines of preclinical medical knowledge. Lectures in the aforementioned subjects are offered on the topics of blood, immunology, the heart, circulation, the gastrointestinal tract, metabolic pathways, energy production, the respiratory system, the endocrine system, the excretory system and the reproductive system as well as their respective embryological development [23]. The third semester lasts 14 weeks and covers lectures, seminars and lab/microscopy sessions. Lectures are organised in weekly blocks reflecting one organ system each. Some organ systems require a detailed description of their biochemistry, while others are better explained through their physiology or histology. For this reason, lecturers from all three subject departments contribute to the lectures in an integrated manner. Learning content of compulsory seminars and lab/microscopy sessions are related to the topics of lectures and enable in-depth learning and individualized consolidation of relevant objectives [24]. At the end of the third semester, medical students have to write a final examination. This examination tests medical students’ proficiency in the aforementioned subjects for each topic. It is a paper-based test of 90 single-answer multiple choice questions.

#### Design of the voluntary examination preparation course for second year students

The above described structure of the third semester was beneficial for the course in that it allowed the tutorial to run in parallel with the lectures. The sessions of the tutorial were offered each Tuesday afternoon, one week after the topic had been treated in the lectures. This continued for the whole course of the semester with a total of 14 sessions. Tuesday afternoons are allocated by the faculty for self-organised learning, so that there were no lectures or compulsory seminars, which would have prohibited the voluntary attendance of the tutorial. Students also had sufficient time to revise the learning objectives of the lectures as well as study independently, prior to reviewing the respective organ system within the tutorial. The tutorial aimed to consolidate contents which students had already encountered.

The sessions of the tutorial were based on the so called sandwich principle [24]. It considers that learning is a highly individual process in which motivation, speed and method vary [24]. Such individuality enables effective collective and individual phases of learning. Collective phases are those in which students are offered the necessary knowledge; they are comparable with frontal instruction [25]. Individual phases challenge the students to process and integrate these new contents. During this phase, students work independently, which further promotes their autonomy. Learning through both phases fosters a positive climate and an increase in long-term academic success.

The tutorial followed this design by encouraging individual presentations and incorporating interactive work. Students prepared and held short presentations on subject related contents (individual learning phase). Such presentations were followed by information given by the tutors (collective learning phase). Presenting students focused on one subtopic of the respective organ system e.g. altitude sickness for the respiratory system. Presentations included 10 minutes of explanation and 5 to 10 minutes of a presenter-guided questions and answers session. Aims were to prompt the understanding through self-explanation for the presenting individual as well as alternate the learning climate for their peers to increase the group attentiveness. Repeating and reformulating ideas deepens processing in the individual knowledge network [26], [27]. Other students also benefit from experiencing different logical approaches. At the same time, students were given the opportunity to present in the German language and practice formulating their thoughts in confident fashion. This is particularly relevant for the oral examination in the first part of the German board examination [28], in which international students perform significantly worse than their German colleagues [9]. Presenters also received constructive feedback and support from their peers, which is a crucial factor in facilitating learning processes [29]. Formulating such questions and responses stimulated students to re-evaluate the contents they had just learned. In addition, students were encouraged to inquire about and discuss uncertainties during the collective phases guided by the tutors. They were asked to create diagrams and do calculations before their peers. The group helped revising these tasks depending on individual ideas. 

#### Peer tutors

In total there were four peer tutors (two female; mean-age 21.8 years (SD 1.3)). From session to session they changed in pairs so that each session of the tutorial for second year international students was held by two tutors together. Two of the tutors were in their third year (KA and CA); one was in his fourth (OA) and one in her fifth year (ACM) of medicine at Heidelberg University. All tutors have an international background (ACM: Canada, KA and OA: Syria, CA: USA). One tutor (ACM) has been holding tutorials for first year international medical students [20] since October 2013. The other three tutors (CA, KA, and OA) joined the team in September 2016 and were assigned the task of conceptualising, preparing, and guiding international medical students in their second year. All tutors were former participants in the tutorial and were recruited as tutors since they showed interest in taking on the job and seemed to be well suited.

#### Participants

The international students at the Heidelberg University Medical Faculty were informed in the second semester of their first year about the continuation of the tutorial for the third semester. Tutorials were voluntary and open for all international students. A detailed description of the participants will be presented in the results section.

#### Quantitative assessment via multiple-choice-questions

At the beginning of each session of the tutorial, participants were asked to answer five multiple-choice-questions on their own (pre-test). These questions were always related to the content as well as learning objectives of following sessions and imitated the question style of the semester examination and the written examination of the first part of the German board examination. All questions appeared in question-type A [30]. Participants were told that these questions were part of the course assessment and additionally serve self-evaluation. At the end of the semester, students were invited to participate in a post-test that served to assess learning achievement during the tutorials and additionally was launched to prepare international students for the upcoming semester examination. Within the post-test all questions were re-used that had already been answered in the pre-tests of the previous sessions (12x5 questions=60 questions). Students’ performances were compared for all of the multiple-choice questions that had been answered at the beginning of each session and the identical corresponding questions that had been answered at the end of the semester to evaluate students’ progress over the course of the semester (pre-post-comparison).

#### Qualitative evaluation of the course by participants

Participants were asked about their thoughts and comments on the course in semi-structured interviews at the end of the semester. Participation in the interview was voluntary. Volunteers who also participated in the multiple-choice test (see quantitative assessment) were provided with book vouchers amounting to 25€ each. Interview questions were developed based on a literature review as well as discussion among a team of experts. The interview guideline was constructed in a semi-structured manner [31], containing mainly open-ended questions, followed by encouraging and clarifying questions if required. The duration of the interviews was between 10 and 20 minutes each; all interviews were conducted in a seminar room at the university hospital. Participants were asked about:

1. reasons for participating in HeiTiMed;
2. what they had learnt in the course, and what were positive or negative impressions of the course;
3. their attitude concerning their own contributions (presentations, demonstrations, discussion), and what was helpful or difficult in this regard, and
4. suggestions to improve the course in future (see the interview guideline in the attachment 1 ).

The individual face-to-face interviews were conducted by one trained interviewer (DH), following the semi-structured interview guideline. Audio recordings were made.

Statistical analysis of pre-post-comparison of multiple-choice-questions and qualitative analysis of semi-structured interviews

For the pre-post-comparison of multiple-choice-questions, only students that participated in the post-test were selected. Due to the fact that not all of them had visited every single tutorial, only the pre-tests they had taken and corresponding post-test multiple-choice-questions could be analysed. To compare pre- and post-results, paired t-tests were calculated. Cronbach’s alpha was calculated as a test of reliability.

For the qualitative data, audio files of the ten interviews were transcribed verbatim and content analysis was undertaken based on principles of qualitative content analysis [32]. First, we conducted an open coding of all the interviews to search for recurring topics. Single or multiple sentences were identified as a code, representing the most elemental unit of meaning [33]. Next, the codes were summarized into relevant themes for each participant, using the software MaxQDA (2010 version, VERBI GmbH, Berlin). As themes recurred across participants, they were then compared and adapted until a number of relevant themes for all participants could be defined. The assignment of respective codes to specific themes was conducted by two independent analysts. They subsequently discussed the coding, and if required, made adjustments once a consensus was reached.

#### Ethics

The ultimate goal of the study was curriculum improvement. The ethics committee of Heidelberg University gave the ethical approval for the study design described (Number: S-535/2016). The study was conducted in accordance with the declaration of Helsinki (revised form, Fortaleza (Brazil), 2013) [34]. All participants gave written informed consent and study participation was voluntary.

## Results

### Student sample

20 (14 female, 6 male; age 21.8±1.8 years) students participated in the tutorial’s sessions. All participants were international students in terms of their nationality and/or the state in which they had gained their highest educational attainment and all of them were in their second year. Table 1 (Tab. 1) gives more detailed information about

1. characteristics of this sample, about
2. students participating in the pre-post-comparison of multiple-choice-questions as well as
3. in the semi-structured interviews at the end of the semester.

#### Pre-post-comparison of multiple-choice-questions

Twelve students participated in the post-test (see Table 1 (Tab. 1) for further characteristics of this sample). To draw a pre-post-comparison of multiple-choice-questions, only those questions of the post-test were included, for which multiple-choice pre-test results were available and accordingly the student visited the respective seminar lesson.

On average, students showed 55.9% (*SD*=11.9%) right answers over all pre-tests and 78.7% (*SD*=11.5%) in the related items of the post-test. The paired t-test showed that pre- and post-results differed significantly from each other (*t*(11)=–8.48, *p*<.001, *d*=1.95). For more details see Table 2 (Tab. 2) and Table 3 (Tab. 3).

#### Test-reliability

Reliability was calculated using Cronbach’s alpha. In the post-test, over all values, also those that were not included into latter analysis (numbers in brackets in Table 2 (Tab. 2)), Cronbach’s alpha was .89.

Due to the fact that many students did not participate in the pre-test, no reliability of it could be calculated because of the missing values.

#### Main categories and themes resulting from qualitative analysis

Qualitative analysis of the transcripts revealed 148 relevant individual statements from participants. From these statements, twelve themes were derived, which were consolidated into four relevant categories. The main categories included (A) reasons for participation, (B) technical and didactic learning experiences, (C) social learning experiences, and (D) individual contributions. Each of these main categories (A to D) contained 2 – 5 themes (i.e. A.1 to A.2).

#### Definition of categories

In the following section, we provide definitions for the themes belonging to the main categories.

##### Category A: Reasons for participation

The category describes different motives among international students that prompted them to participate in the course and includes two relevant themes. The theme “Positive experiences in the first year” (A.1) contains students’ statements about very helpful experiences with the tutorial already in their first year of studies. In most cases they did not really have to decide whether to attend the course or not since they already had got used to going there. “To be in contact with other international students” (A.2) envelopes the idea of coming into contact with other international students in a similar situation. For more details see Table 4 (Tab. 4).

##### Category B: Technical and didactic learning experiences

The second category highlights students’ experiences in terms of technical and didactic approaches which they were able to gain within the tutorial. Five relevant themes emerged. The theme “Enhanced understanding of complex issues” (B.1) includes international students’ improved insight into complex and complicated issues. Within these aspects, they highlight tutors’ use of language which is more accessible to them than the lectures speech habits facilitating their understanding as well as the fact that one focus in the tutorial was to get to know the complex interrelationships of different topics. “Focussing on the topics that are relevant for exams” (B.2) describes students’ benefit from focussing on topics relevant for passing the exam. The theme “Selected teaching materials and tasks in class” (B.3) summarises participants’ statements regarding the materials provided by the tutors as part of the tutorial giving them better opportunities to prepare for the exam. “General revision of topics” (B.4) describes how participants could benefit from the continuous repetition of lesson content in combination with explanations and the possibility to pose questions. Finally, “Interactive elements of teaching” (B.5) illustrates students’ learning experience that interactive elements within the tutorial can be superior to a strategy of mindless memorization. More detailed information is given in Table 5 (Tab. 5).

##### Category C: Social learning experiences

This category illuminates participants’ social experiences which they were able to gain within the tutorial. Three relevant themes could be identified. The theme “The tutorial as a particular setting” (C.1) describes the fact that the tutorial has more characteristics of a seminar than of a lecture. Only a small, homogenous group of international students are meeting each other. Here, they can be themselves, can ask questions and make mistakes without fear of embarrassment. “The special role of the tutors” (C.2) highlights tutors’ extraordinary commitment as well as their characters. They are perceived as really motivated and interested in teaching. Besides, they are recognised to be familiar with international students’ situation because of their own ethnic backgrounds. Finally, the theme “Everyone should understand everything” (C.3) describes the fact that within the tutorial issues are discussed as long as every participant understands everything which is experienced as helpful. Further information is displayed in Table 6 (Tab. 6).

##### Category D: Individual contributions

In this category, issues relating to students’ individual contributions are reported. The category consists of two themes. The theme “Previous perception” (D.1) summarises statements in which students express how good this idea of individual presentation would be. It would be so helpful to practice such frightening tasks in a safe place like the tutorial. However, “The preparation was too time consuming” (D.2) highlights students’ refusal to actually perform this task. They argue that the third semester would be so stressful anyway that they had skipped this task because it was voluntarily. More detailed information is given in Table 7 (Tab. 7).

## Discussion

The aim of the study was to determine whether an innovative course for international second year medical students is a well-accepted and effective method to prepare such students for the final examination at the end of the third semester. We wanted to investigate whether participants in the tutorial improve their results over the period of one semester from weekly multiple-choice pre-tests to a multiple-choice post-test at the end of the semester. Furthermore, we wanted to find out whether participants experience the newly created course-offer as beneficial. In supporting international students, tutorials are an often used format among the German medical faculties [35]. However, so far to the best of our knowledge no result evaluations of such tutorials have been conducted.

The pre-post-comparison of multiple-choice-questions showed a clear picture. In the post-test, students performed significantly better than in the pre-tests conducted throughout the weekly sessions of the tutorial. While in the pre-tests only 55.9% of the multiple-choice questions could be answered correctly, in the post-test the number of correct answers increased to 78.7%. All students investigated improved themselves from pre- to post-test; every single student seemed to be able to pass the upcoming final examination at the end of the third semester. Although the highly improved results in the post-test might not be too surprising since students might also have started to learn for the final examination by that time, this improvement can still be seen as success of the tutorial. However, in order to really assess tutorial’s effectiveness a randomised controlled trial should be conducted including a control group not participating in the tutorial.

The point that only twelve out of 20 students participated in the post-test might be due to the fact that at the end of the semester most students were involved in preparing for the final examination and saw no reason for participating in this post-test. 

The qualitative interviews, conducted at the end of the semester, gave a detailed picture of the participants’ experiences with the course. All of them were highly satisfied with the course, indicating a range of benefits including technical and didactic benefits as well as social learning experiences. However, concerning the individual contributions, most students prompted that although these individual presentations seemed to be a good idea on the one hand, they would have been too much effort on the other hand.

Regarding participants’ motives to join the course in the third semester of their studies, the content analysis revealed that positive experiences with HeiTiMed in the first two semesters were one of the most relevant factors. Since they had experienced the tutorial as very helpful before, students did not really have to take a decision whether to join the tutorial or not, they just kept on going there. The lasting contact to other international students was another crucial point underlying students’ motives to join the course. The above mentioned lack of social contacts [6] might be an explanation for this. The international students perhaps have made friends with other participants in the tutorial which might have become another important reason for attending the tutorial. Group cohesion seems to play an important role in such a small groups [36]. International students, reporting insufficient support [5] as well as a loss of social contacts [6], can find a place or a feeling of belonging. They can meet fellow sufferers, can protect and support each other.

With respect to technical and didactic learning experiences, the study revealed that the tutorial enabled students’ enhanced understanding of complex issues. They did not only want to learn study contents in an empty-headed manner, they were interested in understanding all the complex interactions. This fact indicates that deep processing seems to promote consolidation and inclusion in an associative knowledge network [27]. Furthermore, by means of tutors’ more informal use of language compared with the lecturers’ technical jargon it was much easier for students to understand even the complicated issues [37]. In addition to this focus on understanding, however, also specification of content as well as the prioritisation on topics relevant for the final examination seemed to be beneficial for students. It was useful for them to actively assess the most relevant exam topics and associated key literature which made them feel better prepared for the final examination. The participants also benefitted from the provided materials like power-point-presentations or illustrations which allowed them a different access to the teaching contents. Moreover, international students even welcomed the general repetition of contents. To listen to the contents twice – first in the lecture, one week later in the tutorial – allowed them to internalise contents better. Finally, the course’s interactive teaching elements seemed to have an effect on some of the students. On the one hand, this helped to loosen the tutorial’s atmosphere; on the other hand, this strategy represents a contrary approach to a learning method of mindless memorization. Following the sandwich method [24], these phases in which the necessary knowledge is presented to students are alternated with phases in which students’ autonomy as well as deeper understanding are fostered through individual contributions.

Regarding social learning experiences, the study revealed that the tutorial’s format was important to students. They appreciated the personal atmosphere of the small group. They knew each other well and so could pose any question or make mistakes without worrying about it. Somehow the tutorial was seen as secret place only the group of international students knew from which gave them a feeling of belonging. Another prominent issue was seen in tutors’ outstanding commitment and effective work. They are seen as role models, counterparts and teachers [38]. Due to tutors’ own migration background social congruency seems to play an important role in the interaction [37]. Finally, the international students saw advantage in that all aspects in the tutorial were discussed until even the last student would have understood them. No one was left behind; the tutorial was experienced as a place where everybody could indeed find help.

With respect to students’ individual contributions, the content analysis revealed a contrary picture in students: While all of them had the opinion that such individual presentations would be a very good idea and a chance to practice this frightening issue, most of them had to confirm that they had not taken this chance due to a too heavy workload throughout the semester. Due to the fact that they were obliged to present in at least two seminars during the semester on top of all the other compulsory tasks they had a feeling of not being able to cope with this requirement. This draws an ambivalent picture: Even while the students have a feeling that it would be very important to them to practice their competencies in lecturing in German language, at the same time it becomes apparent that international students already seem to be very busy with other study-related activities and therefore tend to refuse to hold a speech within the tutorial.

It might be speculated if such tutorials contribute to international students’ support in the following ways: Due to the fact that international students perform worse than their German colleagues – especially in the very first semesters of their studies [9] – they might be worried about not passing examinations and to master their studies in general. Within the tutorial, tutors with migration backgrounds themselves can function as excellent role models [39], can remove students’ fears and can teach them what is important to finally pass the upcoming examinations [20]. The existing social as well as cognitive congruence between students and tutors [21] seems to have a great impact here: Because of their comparable social roles tutors can act more empathetic with students’ worries. And students seem to benefit from the more informal way of teaching which uses less technical terms and contributes more to an understanding. Furthermore, in the tutorial international students can find a place in which they meet peers that are somehow in a similar situation: Far away from home in a foreign country. Establishing such personal relationships with like-minded and friendly peers seems to be an important factor, comparable to a social interface through which international students are able to step out of anonymity. Another important issue is that within the tutorial international students receive a formative feedback about their individual performance level which shows them existing strengths and weaknesses. Besides, the post-test can be seen as a simulation of the final examination which can contribute to a better adaptation as well as a possible overcoming of fears.

### Limitations

The quantitative evaluation was limited concerning the following issues: First, the low sample size has to be taken into account. Furthermore, pre-tests were carried out right before subsequent sessions dealt with the relevant contents. At this point, students might have prepared differently: some preferred to review contents immediately after the lecture and before the tutorial session while others did so later. The post-tests were taken at the end of the semester after all contents had been reviewed. The evaluations were taken right before the semester multiple-choice examination had to be written. Therefore, it might be seen as not surprising that students performed better in the post- than in the pre-evaluation. Also, participants’ improvement is not necessarily linked to the tutorial. To really assess its effectiveness a controlled trial with a second group not participating in the tutorial would be needed. Another limiting aspect lies in the fact that we had no possibility to compare the results of pre- and post-tests with the results of the final examination at the end of the third semester due to data security reasons. Therefore, it might be possible that students have improved from pre- to post-test but not from these results to the real examination. However, as pre- and post-test-questions imitated the question style of the semester examination these results at least somehow should reflect the “real” results. 

The qualitative part of our study was limited by the number of participants. Another limitation occurred due to interviews’ shortness that allowed only provisional statements. Participation in interviews at the end of the semester was voluntary, and this may possibly have led to biases in our analysis. Volunteering students received book vouchers for their participation in the study. These provisions could also have biased the results. Finally, although the analysis was performed according to principles of qualitative content analysis [32] and was verified by a second analyst, the evaluation might still be considered more subjective than a quantitative analysis.

## Conclusion

To the best of our knowledge, the current study is the first to examine a peer-guided tutorial for second year international medical students. This specific group of students suffers from distress, insufficient support as well as a loss of social contacts. Besides, international medical students perform worse in written, oral and practical examinations; show a prolonged duration of study as well as higher drop-out rates than their German colleagues. Our peer-guided tutorial for second year international medical students showed to be a well-accepted method to support them on a voluntary basis. Furthermore, results of the pre-post-comparison of multiple-choice-questions give a first indication on the effectiveness of the tutorial. However, these results are limited in terms of small sample size and low representativeness. Therefore, future research should aim at conducting a randomised controlled trial on the effectiveness of such a tutorial. 

## Authors’ contributions

CN and DH conceived the study. DH, KA, OA, CA, ACM, WH, and CN participated in the design of the study. KA, OA, CA, and ACM organized the tutorial and helped in coordinating the study. DH conducted the semi-structured interviews. DH and CN carried out the qualitative analysis and finally drafted the manuscript. All authors read and approved the final manuscript. 

## Competing interests

The authors declare that they have no competing interests. 

## Supplementary Material

Interview guideline for international second year students

## Figures and Tables

**Table 1:**
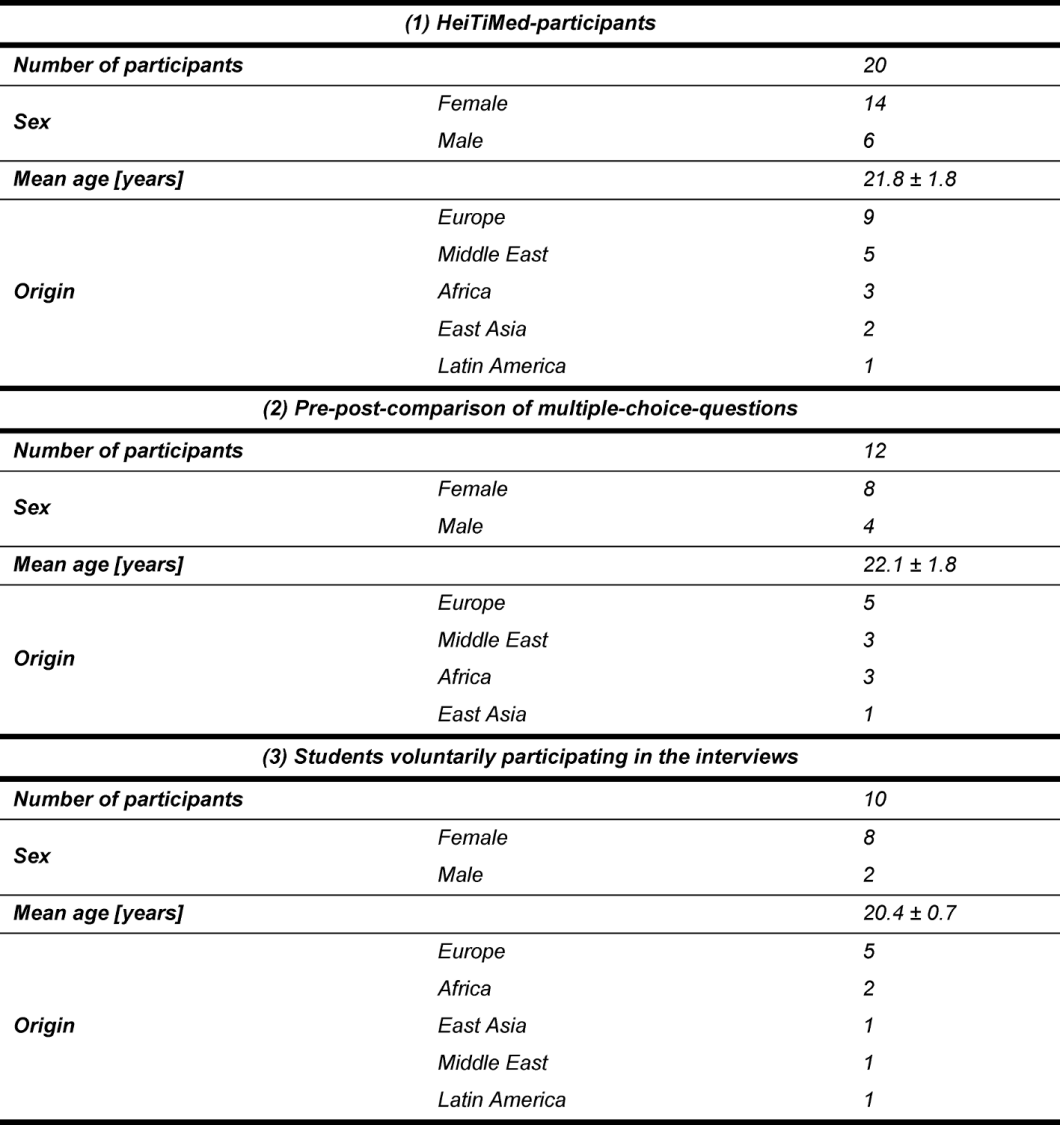
Characteristics of the (1) HeiTiMed-participants, the (2) students participating in the pre-post-comparison of multiple-choice-questions as well as in the (3) semi-structured interviews at the end of the semester

**Table 2:**
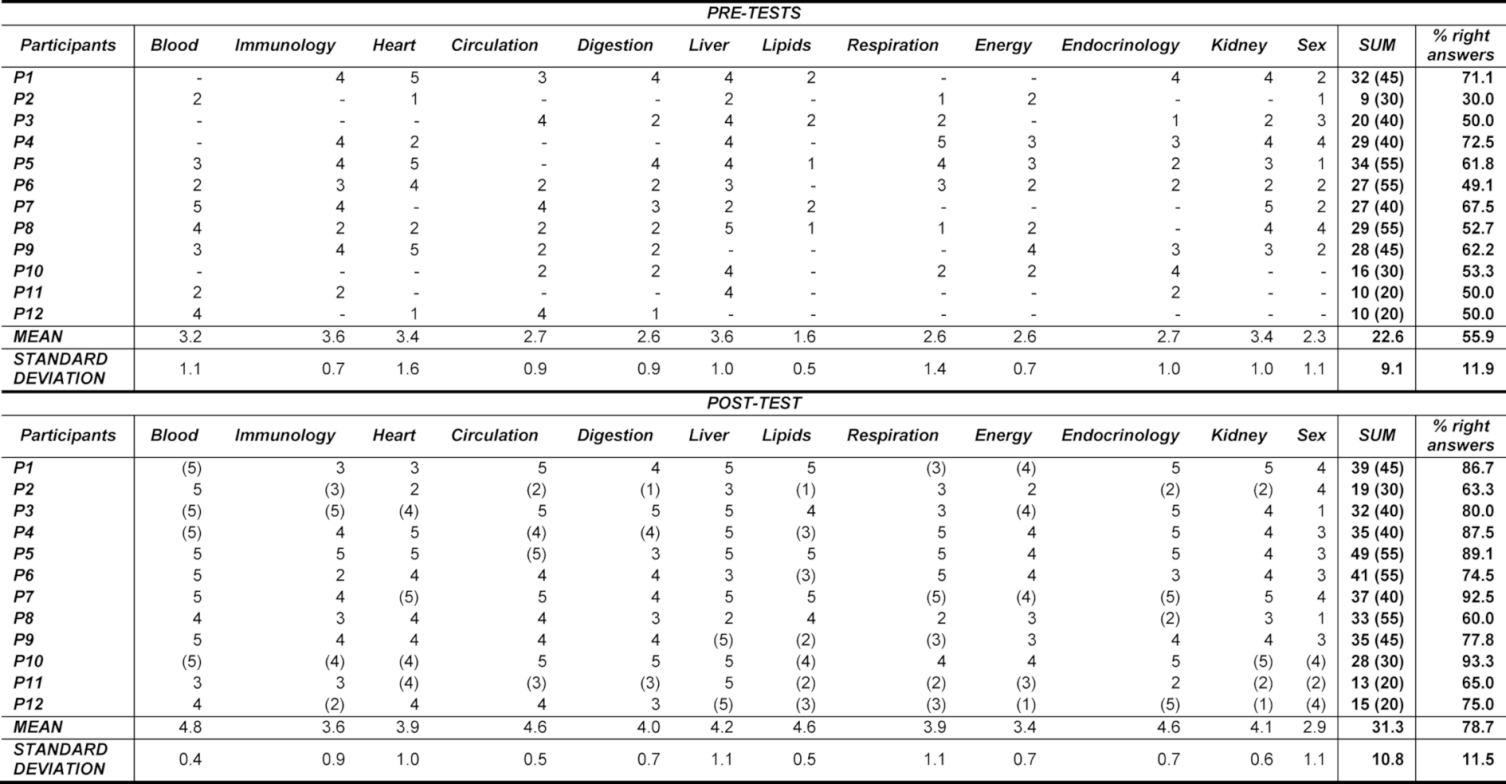
Pre-post-comparison of multiple-choice-questions. The scores given correspond to the number of correct answers of five questions each. Within the post-test, only such scores related to the pre-tests were calculated. All the other ones are listed in brackets and were not part of the analysis.

**Table 3:**
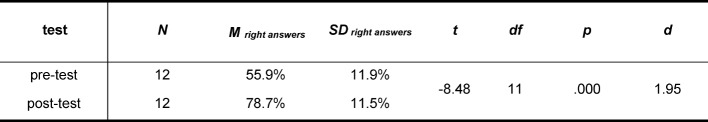
t-test for the differences between pre- and post-test results

**Table 4:**
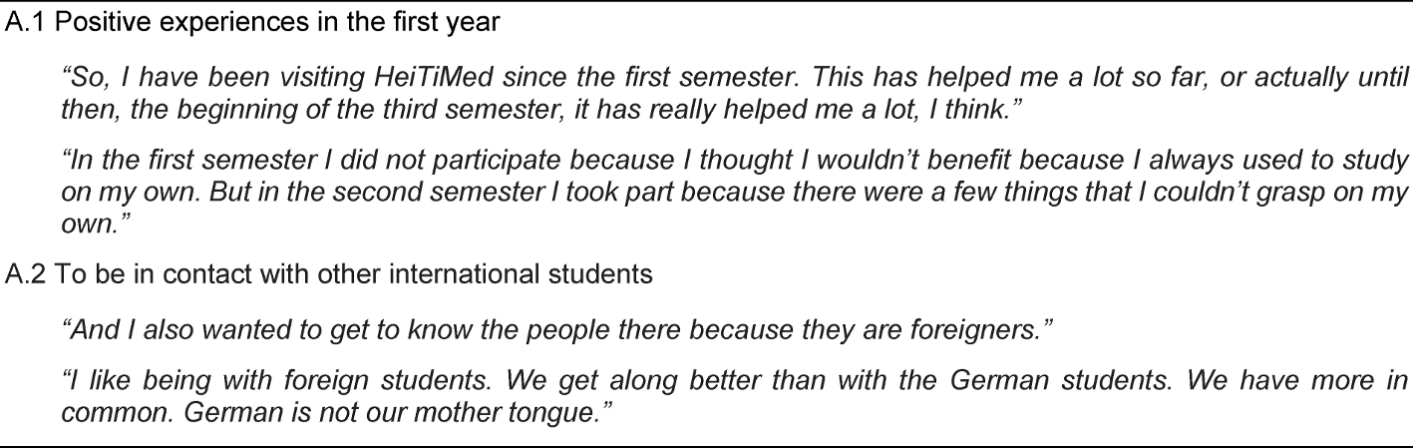
Main Category A – Reasons for participation: Why did the international students choose to participate in HeiTiMed?

**Table 5:**
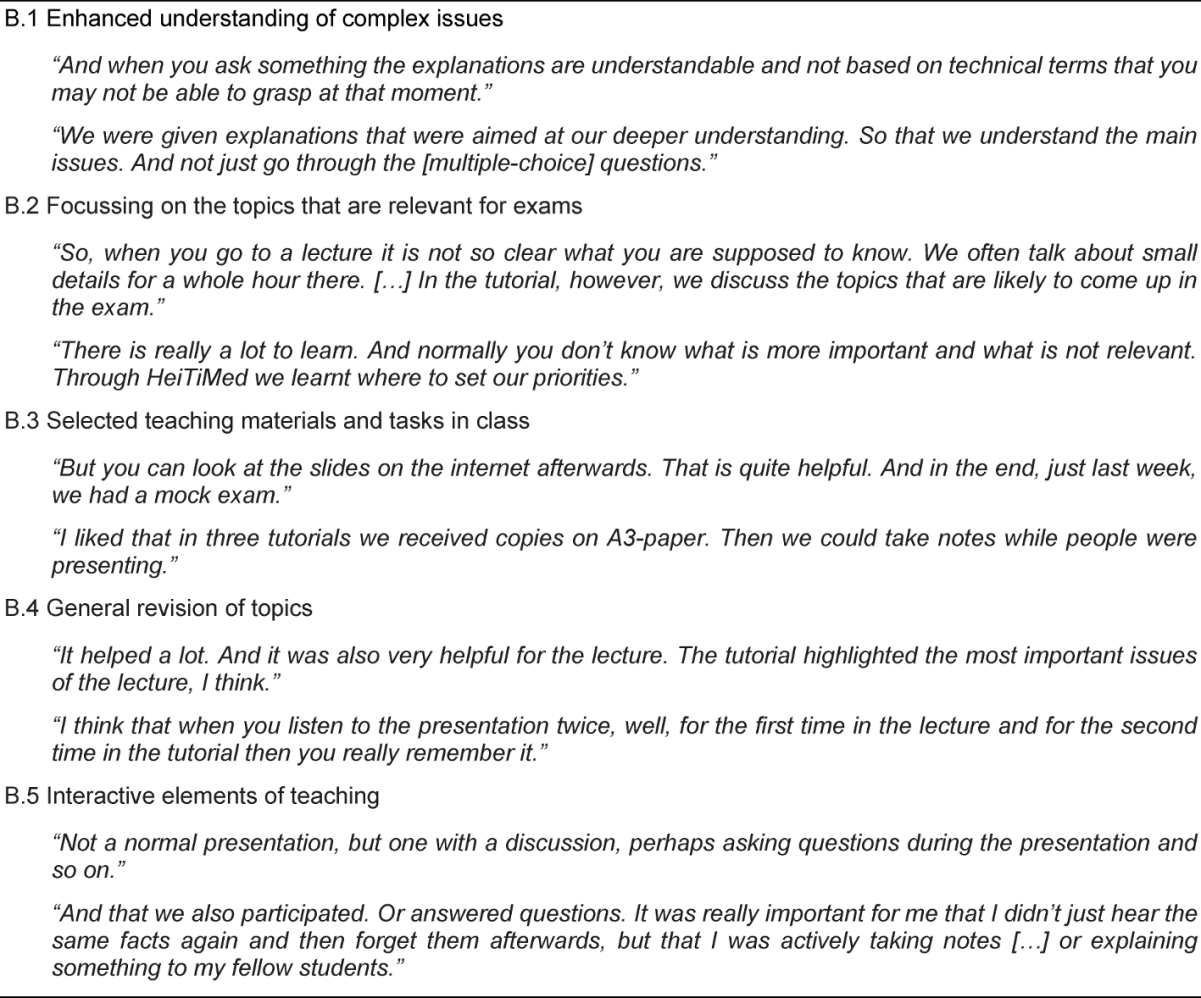
Main Category B – Technical and didactic learning experiences: What kind of technical or didactic experiences did the students have in the tutorial?

**Table 6:**
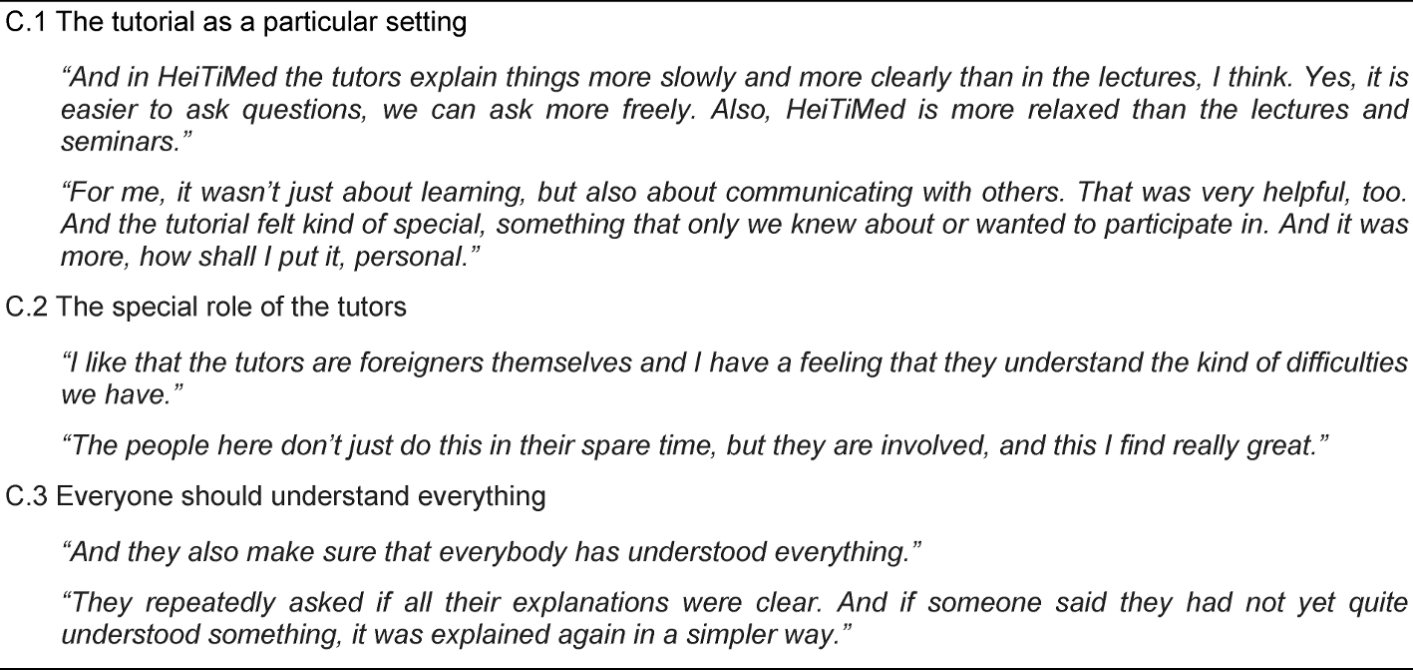
Main Category C – Social learning experiences: What kind of social experiences did the students gain during their participation in the tutorial?

**Table 7:**
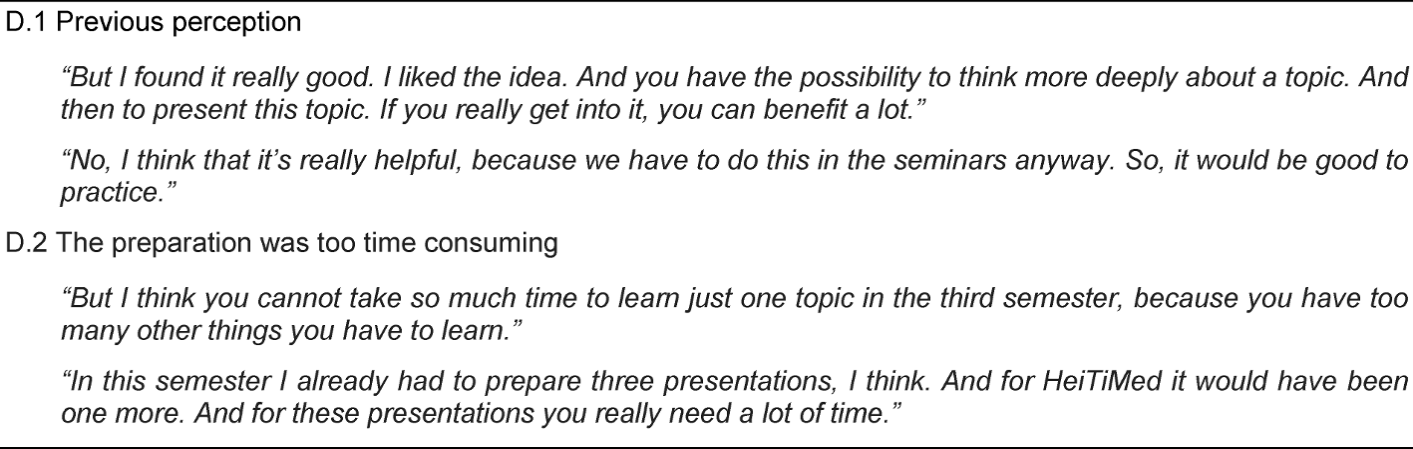
Main Category D – Individual contributions: What did the students experience when they were invited to give short presentations in front of the group?
