# Supplementary material for: Interactive peer-guided examination preparation course for second-year international full-time medical students: quantitative and qualitative evaluation
Source: GMS J Med Educ. 2018 Nov 30;35(5):Doc57. doi: 10.3205/zma001203 (PMC6326399; doi:10.3205/zma001203)
Supplement: Interview guideline for international second year students [file JME-35-57-s-001.pdf]

OPENING:

- What were your reasons for attending HeiTiMed in the past semester?

INTRODUCTORY:

- What did you learn within the context of HeiTiMed?
- What was positive, what was negative?

KEY QUESTIONS:

- Which didactic approaches did you get to know in HeiTiMed? Which did you find especially helpful? What difficulties did you encounter?
- Did you find it helpful to contribute to the tutorial with your own presentation?
- Was HeiTiMed helpful in regard to your studies?

ENDING:

- Where do you see room for improvement in the HeiTiMed program? What should be changed?
